# Computational Biology: Moving into the Future One Click at a Time

**DOI:** 10.1371/journal.pcbi.1004323

**Published:** 2015-06-24

**Authors:** Christiana N. Fogg, Diane E. Kovats

**Affiliations:** 1 Freelance Science Writer, Kensington, Maryland, United States of America; 2 Executive Director, International Society for Computational Biology, La Jolla, California, United States of America

## Overview

Computational biology has grown and matured into a discipline at the heart of biological research. In honor of the tenth anniversary of *PLOS Computational Biology*, Phil Bourne, Win Hide, Janet Kelso, Scott Markel, Ruth Nussinov, and Janet Thornton shared their memories of the heady beginnings of computational biology and their thoughts on the field’s promising and provocative future.

## Philip E. Bourne

**Fig 1:**
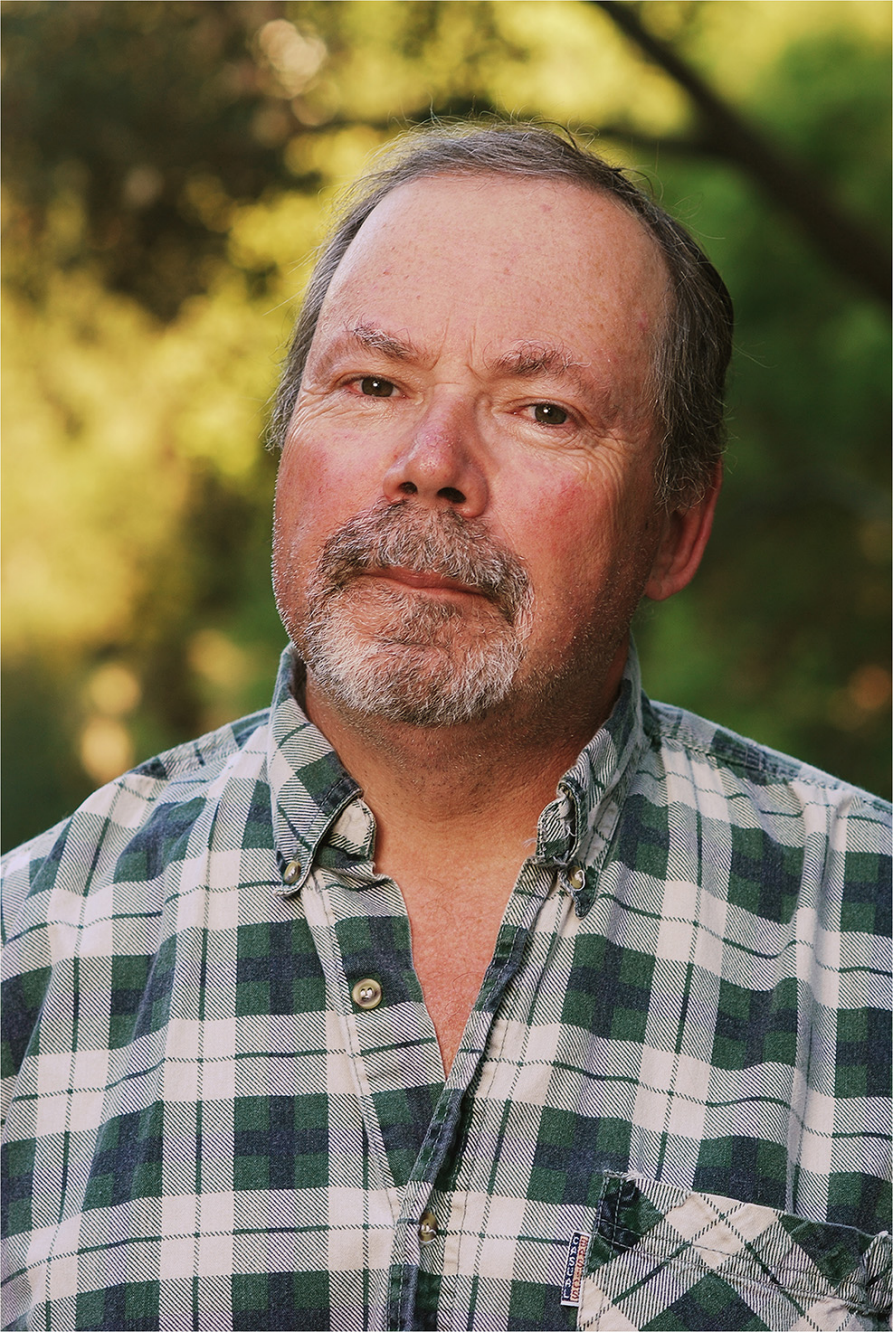
Philip E. Bourne. Associate director for data science, NIH.

Philip Bourne (Fig 1) began his scientific career in the wet lab, like many of his computational biology contemporaries. He earned his PhD in physical chemistry from the Flinders University of South Australia and pursued postdoctoral training at the University of Sheffield, where he began studying protein structure. Bourne accepted his first academic position in 1995 in the Department of Pharmacology at the University of California, San Diego (UCSD), rose to the rank of professor, and was associate vice chancellor for Innovation and Industry Alliances of the Office of Research Affairs. During his time at UCSD, he built a broad research program that used bioinformatics and systems biology to examine protein structure and function, evolution, drug discovery, disease, and immunology. Bourne also developed the Research Collaboratory for Structural Bioinformatics (RCSB) Protein Data Bank (PDB) and Immune Epitope Database (IEDB), which have become valuable data resources for the research community. In 2014, Bourne accepted the newly created position of associate director for data science (ADDS) at the National Institutes of Health (NIH), and he has been tasked with leading an NIH-wide initiative to better utilize the vast and growing collections of biomedical data in more effective and innovative ways.

Bourne has been deeply involved with the International Society for Computational Biology (ISCB) throughout his career and is the founding editor-in-chief (EIC) of *PLOS Computational Biology*, an official journal of ISCB. He has been a firm believer in open access to scientific literature and the effective dissemination of data and results, for which *PLOS Computational Biology* is an exemplary model. Bourne believes that open access is more than just the ability to read free articles, and he said, “The future is using this content in really effective ways.” He referenced an article he cowrote with J. Lynn Fink and Mark Gerstein in 2008, titled “Open Access: Taking Full Advantage of the Content,” which argued that the full potential of open access has not been realized, as “no killer apps” exist that troll the literature and cross-reference other databases to come up with new discoveries [1]. Bourne believes that the scientific literature will become more and more open in this digital age and that new tools will be developed to harness the potential of this expansive information treasure chest.

Beyond scientific literature, Bourne sees data as a powerful catalyst that is often trapped in individual servers or hardware from defunct projects. As ADDS, he is steering the development of the Commons that he sees as a virtual space in which data sets, tools, and results can be stored, indexed, and accessed. Bourne anticipates that the Commons can be a way for tools and data to live on and benefit other scientists or, in other cases, act as a way to flag problematic or unusable contributions. He is hopeful this new approach can change the way biomedical research data is used, believing that “it offers the opportunity for serendipitous discovery.” Software tools that scientists store in the Commons will have better exposure and offer new opportunities for their use and attribution.

Bourne was in attendance as President Obama launched the President’s Precision Medicine Initiative on January 30, 2015, at the White House. This initiative aims to revolutionize medicine by harnessing information about an individual’s genome, environment, and lifestyle, and it will support research projects focused on transforming cancer treatment [2]. A bold long-term goal of the initiative is to create a cohort of 1 million American volunteers who will share their genetic and health information. Bourne sees this potentially revolutionary project as a powerful way to promote collaboration between computational biologists working on basic research problems and medical information scientists focused more on clinical and electronic health record information analysis.

Collaboration will be paramount to the success of projects like the Precision Medicine Initiative, and Bourne has observed how scientists are taking novel approaches to form collaborations in this increasingly connected world. He sees communities self-organizing and springing up spontaneously, and some have become influential and respected advocates for research and data sharing, like the Global Alliance for Genomics and Health and the Research Data Alliance. He said, “These are groups of volunteers [who are] funded to do something else but see [the] value of doing things together.” These communities and alliances offer new ways for scientists with shared research interests to come together both in person and virtually and may offer valuable lessons to scientific societies wanting to remain relevant and useful to their members.

Scientific communities are often grounded in shared ideas, and these ideas can be captured in a community’s specialized publications. During his tenure as EIC of *PLOS Computational Biology*, Bourne began the “Ten Simple Rules” collection, which has become one of the most-viewed article collections in any journal, with over 1 million views. This collection has become a treasured source of ideas and information for the computational biology community and has been relevant and helpful to biomedical scientists and trainees from many disciplines. Bourne considers the popularity of these articles an indicator of the information trainees and scientists are seeking but don’t get during their training. He thinks of the “Ten Simple Rules” articles as starting points and hopes that they genuinely help readers find information or guidance. If not, their entertainment value is remarkably therapeutic for the beleaguered scientific masses.

Bourne is looking forward to the next ten years and beyond as computational biology becomes ever more entwined with biomedical research and medicine.

## Winston (Win) Hide

**Fig 2:**
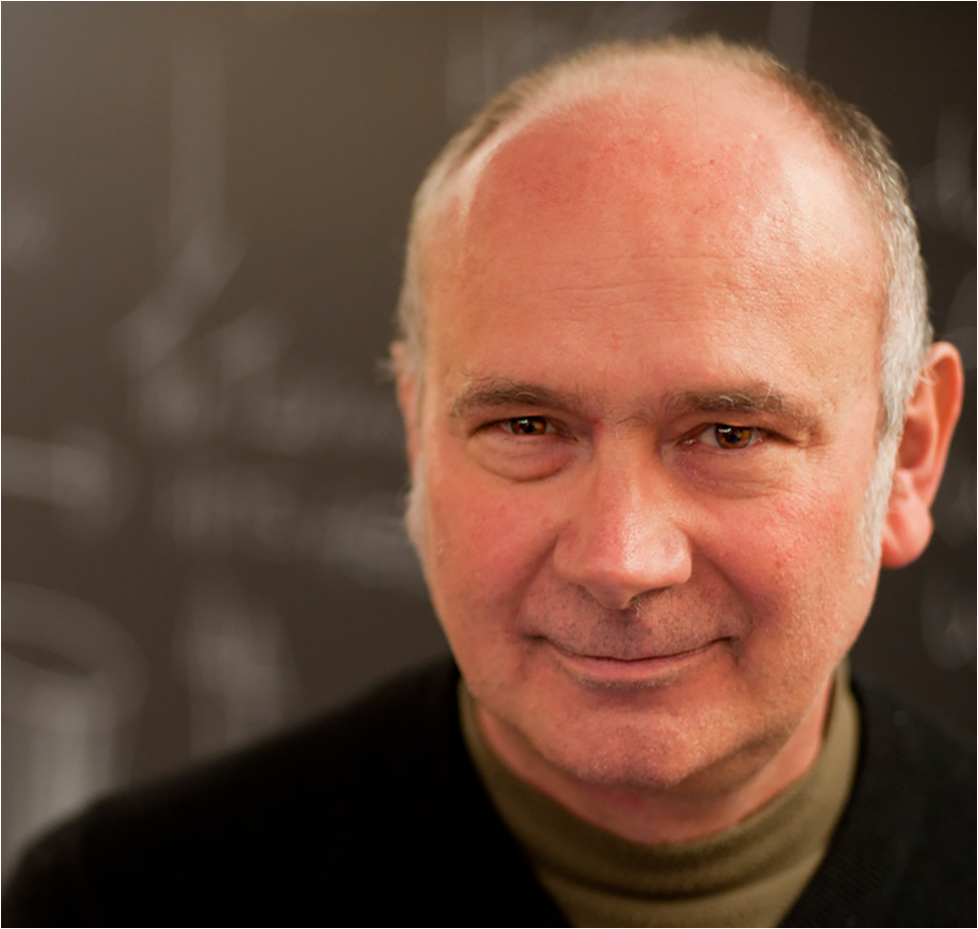
Winston (Win) Hide. Professor of Computational Biology and Bioinformatics, Sheffield Institute for Translational Neuroscience, the University of Sheffield, United Kingdom.

Win Hide (Fig 2) has witnessed the transformation of biological research firsthand, from his early wet-lab training in molecular genetics to his present-day research using computational approaches to understand neurodegenerative diseases. Hide graduated from Temple University with a PhD in molecular genetics, and after his postdoctoral training and time spent in Silicon Valley, he founded the South African National Bioinformatics Institute in 1996. He accepted a position at the Harvard School of Public Health in 2008 and became director of the Harvard Stem Cell Institute Center for Stem Cell Bioinformatics. In 2014, Hide accepted a position at the Sheffield Institute for Translational Neuroscience, University of Sheffield, and became a professor of Computational Biology and Bioinformatics.

Hide considers the drive to sequence the human genome in the 1990s as a major point at which computational biology transformed into a research field. He recalls remarks made around that time, by Lee Hood of the Institute for Systems Biology, contending that biology was becoming a data science. By 1998, SmithKline Beecham had organized the largest corporate bioinformatics department, headed by computational linguist David Searls, and this investment brought new attention to the power and scale of computational biology [3].

Biomedical researchers are beginning to acknowledge that biology is changing from a hypothesis-driven science to a data-driven science. But Hide thinks this shift is causing an uncomfortable and unsustainable tension between scientists working in these different realms. He has witnessed the tendency of hypothesis-driven experimentalists to pick the data they want to use, and these choices work against the innate objectivity of data-driven analysis. “People choose the pieces of data-driven science that make sense to them,” he said. “We haven’t reached the point in computational biology to judge how right we are. We’ve reached the probability but not summation of how good a model is in data-driven science. So we leave it to the biologists to decide on the pieces they know might be right and move forward.”

Hide sees a significant and urgent need for the convergence of the computational biology domains, including text mining, crowdsourcing, algorithmics, systems biology, and large surveys, to arrive at how correct models are by using machine learning. He acknowledges that these sorts of projects are difficult to take on but are likely the only way to arrive at models that are actionable.

Biologists across the board need to become more comfortable with data analysis and coding. Hide highlighted a talk given by Sean Eddy of the Howard Hughes Medical Institute at the “High Throughput Sequencing for Neuroscience” meeting in 2014 that was a gentle but compelling challenge to experimental biologists to reclaim their data analysis [4]. Eddy said, “We are not confident in our ability to analyze our own data. Biology is struggling to deal with the volume and complexity of data that sequencing generates. So far our solution has been to outsource our analysis to bioinformaticians.” He spoke about the widespread outsourcing of sequencing analysis to bioinformatics core facilities. “It is true that sequencing generates a lot of data, and it is currently true that the skills needed to do sequencing data analysis are specialized and in short supply,” he said. “What I want to tell you, though, is that those data analysis skills are easily acquired by biologists, that they must be acquired by biologists, and that that they will be. We need to rethink how we’re doing bioinformatics.” He urged biologists to learn scripting, saying “The most important thing I want you to take away from this talk tonight is that writing scripts in Perl or Python is both essential and easy, like learning to pipette. Writing a script is not software programming. To write scripts, you do not need to take courses in computer science or computer engineering. Any biologist can write a Perl script.”

Hide also sees a great need for computational biologists to be trained to collaborate better. He has witnessed the increasingly collaborative and multidisciplinary nature of biological and biomedical research and contends that computational approaches are becoming a fundamental part of collaborations. In the future, Hide expects that some of the strongest and most successful computational biologists will be specialists in particular fields (e.g., machine learning, semantic web) or domains (e.g., cancer, neuroscience) that excel at reaching across disciplines in nonthreatening and productive ways.

Many experimental biologists first became acquainted with computational biology and bioinformatics through collaborations with researchers running core facilities. Most computational biologists recognize that providing service work is an unavoidable part of their job, but this work is often not appropriately recognized or attributed. Hide believes that computational biologists, collaborators, administrators, and funding agencies must better differentiate between work done for research or as a form of service. Recognition of service work is critical to ensuring that core facilities remain a vibrant part of the research infrastructure and can attract highly skilled computational biologists.

## Janet Kelso

**Fig 3:**
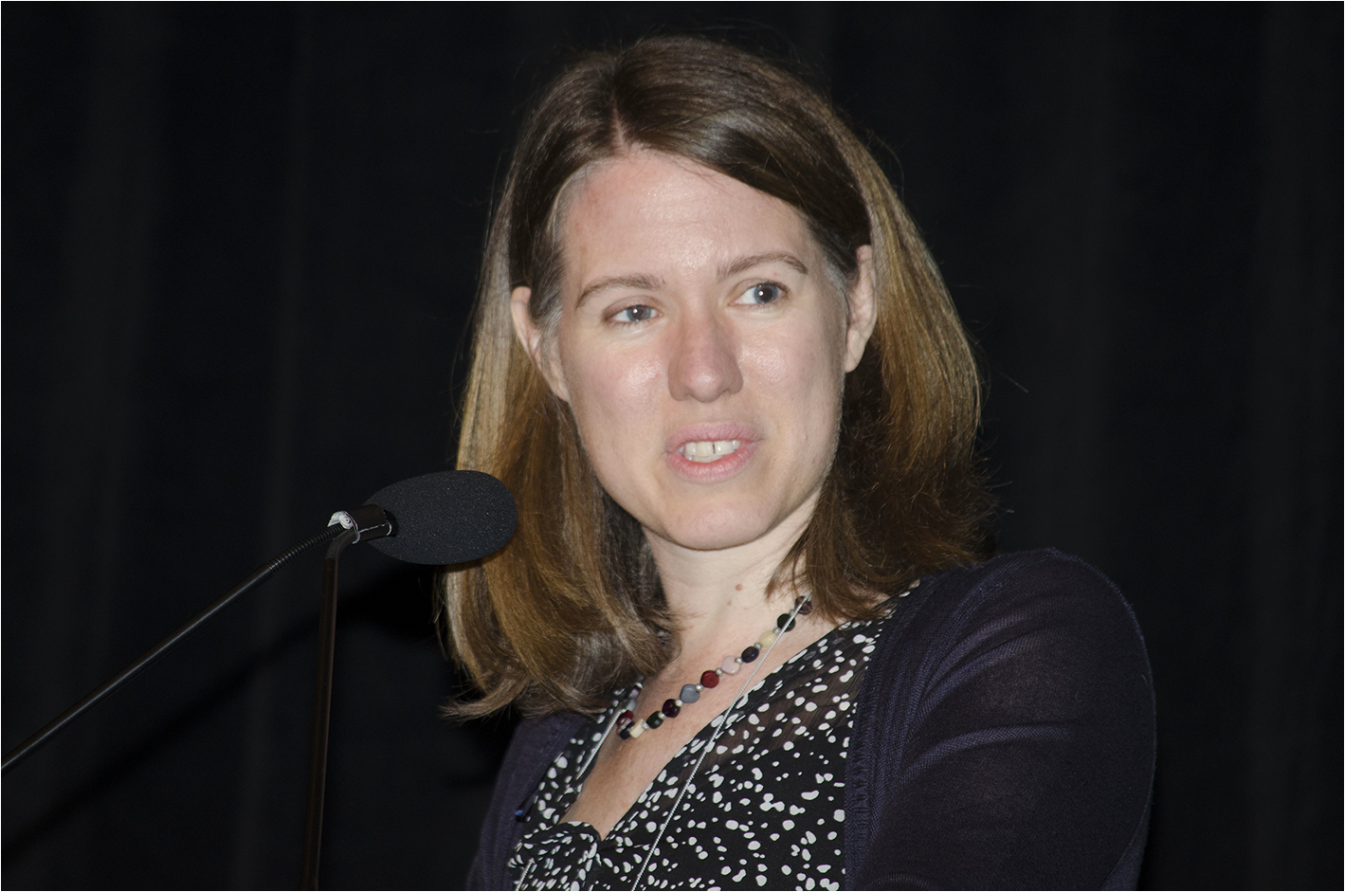
Janet Kelso. Bioinformatics research group leader, Max-Planck Institute for Evolutionary Anthropology, Leipzig, Germany.

Janet Kelso (Fig 3) is working at the cutting edge of computational biology with some of the world’s most ancient DNA. Her research interests include human genetics and genome evolution, with a particular interest in the ancestry of modern *Homo sapiens* and their ancestors, including the extinct Neanderthal species.

Kelso pursued her PhD studies in bioinformatics in the early 2000s under the guidance of Winston Hide at the South African National Bioinformatics Institute. She has watched firsthand as the era of personal genome sequencing has become a reality. “It has become possible in the last eight years to sequence whole genomes so rapidly and inexpensively that the sequencing of the genomes of individual people is now possible,” she said. Kelso thinks the lowered cost and rapid speed of whole genome sequencing will transform our knowledge of human genetics and change the way that medicine is practiced. She sees a prodigious job ahead for computational biologists when it comes to what we do with this data and how we interpret it. “Reference databases created by computational biologists, with information like sequence variants, will capture the effects of ordinary variation in our genomes on our phenotype—how we get sick, how we age, how we metabolize pharmaceutical drugs,” she explained. “This information will be used for diagnosis and will be important for tailoring treatments to individuals.” Kelso expects there will be insight into how genetic variations impact the effect of different treatments, even though these variations may have nothing to do with the disease itself.

As personal genomics becomes a reality, Kelso thinks computational biologists will have to consider that the public will want access to their tools and resources. “The computational biologist’s role is to provide good resources and tools that allow both biomedical researchers and ordinary people to understand and interpret their genome sequence data. It’s a really hard problem, to go from sequence variants in a genome of 3 billion bases to understanding the effects they may have on how long you live or if you develop a disease.”

Kelso sees that computational biology tools will also have immense value in fields other than human genetics. “Many of the tools we develop can be applied in other domains such as agriculture,” she explained. “For example, how do variations in a plant genome allow it to respond to environment, and what additional nutrients do you need to provide to optimize crop production? Similar computational biology tools can be applied in these different systems.”

Kelso considers many of the technical improvements in the field to have been among the major developments in computational biology over the last decade. She explained, “To me, the biggest contributions of computational biology are developments in how to store and annotate data, how to mine that data and to visualize large quantities of biological data. Our ability to integrate large volumes of data and to extract meaningful information and knowledge from that is a huge contribution and has moved the field forward substantially.”

From Kelso’s perspective, she thinks students are now more comfortable with integrating computation into their training and research. “Compared with ten years ago, lab-oriented students are becoming more skilled in bioinformatics. There will always be a place for specialists in both computational and molecular biology, but there is a larger zone in the middle now where people from these different disciplines understand each other.” Kelso has observed that many students now realize that you can’t be a molecular biologist and not know anything about informatics. “Students who come into our program now spend a lot of time learning bioinformatics and are able to work on reasonably sized data sets.”

Kelso is optimistic about the future of computational biology: “Computational biology is now a mature discipline that has cemented itself as integral to modern biology. As we enter a period of unparalleled data accumulation and analysis, computational biology will undoubtedly continue to contribute to important advances in our understanding of molecular systems.”

## Scott Markel

**Fig 4:**
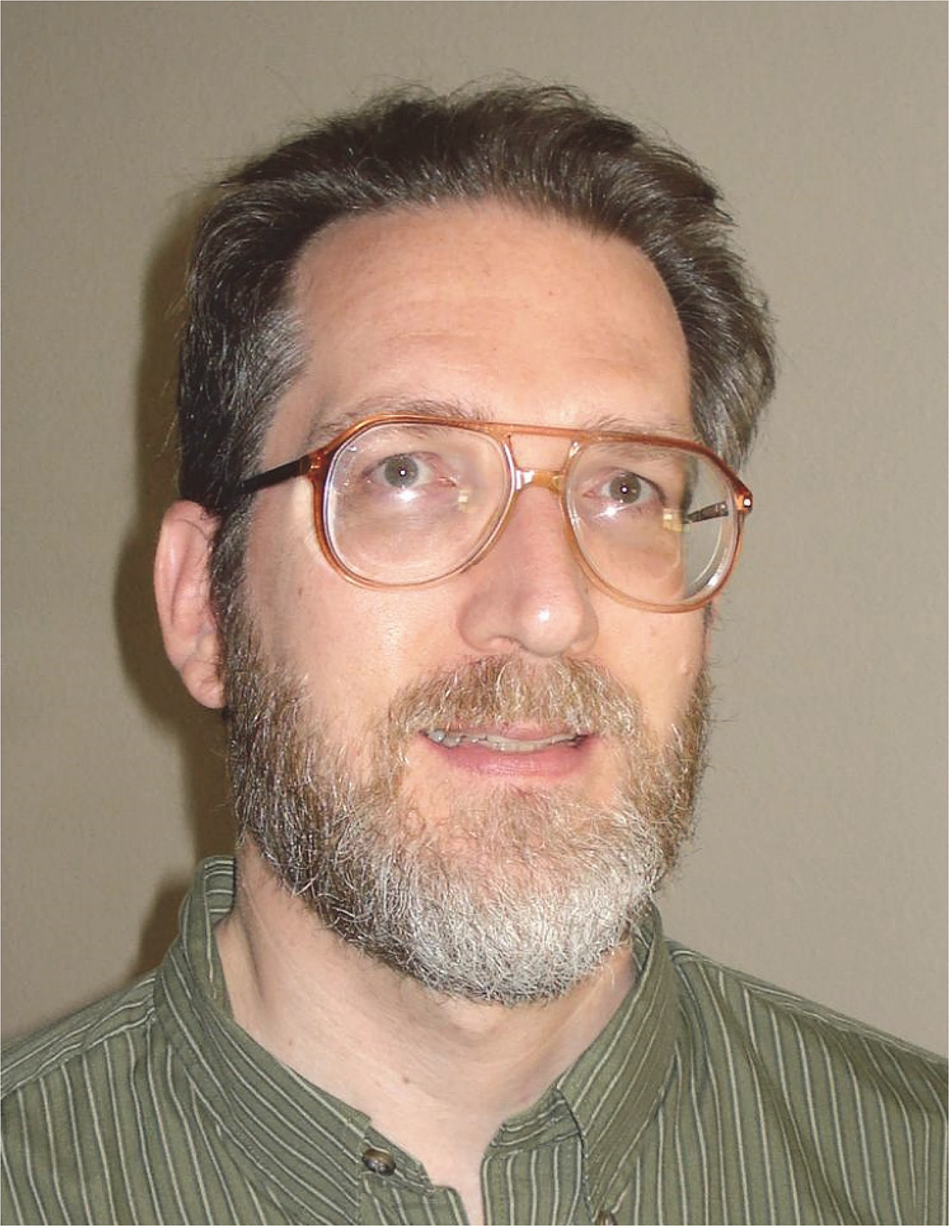
Scott Markel. Secretary, ISCB. BIOVIA principal bioinformatics architect at Dassault Systèmes.

Scott Markel (Fig 4) has spent most of his career working as a software developer in industry. He pursued his PhD in mathematics at the University of Wisconsin–Madison, and like many of his contemporaries, he discovered that he could apply his degree to bioinformatics software development. He said, “I have probably made more of a career in industry than others have by leveraging open source tools, giving back to that community where and when I can.” Indeed, it was the culture of open source software supported by ISCB, especially members of the Bioinformatics Open Source Conference (BOSC) community, which drew Markel to the Society.

Markel, like many of his ISCB colleagues, considers the sequencing of the human genome as a major research landmark for computational biology and a powerful driver of the technologies and software developed over the last two decades for sequencing and genomics. Sequencing technology continues to become cheaper, faster, and more portable. Next-generation sequencing (NGS) technology has been adapted widely over the last five years, but Markel also sees increasing use of newer technologies, like those being developed by Oxford Nanopore, which will offer longer sequence reads. Markel has observed that researchers and bioinformatics information technology (IT) support staff are faced with the challenges of storing vast amounts of digital data and are shifting their mind-set in this era of technological flux. He said, “As sequencing gets cheaper, it’s better not to save all the data—just run the sequence analysis again.”

As an industry-oriented computational biologist, Markel has a different view of how software is developed and used. Markel has learned how to listen to clients’ needs while also balancing out what kind of product can be built, sold, and maintained. “Customers don’t want something like BLAST [Basic Local Alignment Search Tool]; they want BLAST. As team sizes get smaller and broader, it’s not worth building something the equivalent of BLAST, which will need maintenance, need to be sold as a product, and users will have to be convinced scientifically…is as good as BLAST.” Markel’s primary product, Pipeline Pilot, is a graphic scientific authoring application that supports data management and analysis. He has observed that clients in biotechnology and pharmaceutical research are working more on biologics, like therapeutic antibodies, and they are handling an increasingly diverse spectrum of data types. Markel has noticed that clients are drawn to software like the new Biotherapeutics Workbench, built for antibody researchers using Pipeline Pilot, because it provides decision support, and he explained, “Lab time is more expensive than doing things computationally. This type of application can identify subsets of candidates that you can take forward.”

Markel’s software development experiences highlight how computational biology is transforming research in industry settings, and he suspects that industry will continue to invest in computational biology-driven technologies. “If you make the programming part easier,” he said, “like being able to modify the workflow by changing settings or deploying a program through a web interface, users are thrilled to be self-enabled. For some people, especially those without a programming background, this is a revelation.”

Computational biology is likely to become a part of routine health care in the future, and Markel suspects that one area we will see this change in is the “internet of things.” Computational biology applications are not limited to research and drug discovery but are already being adapted for clinical use, like implantable devices, home health monitoring, and diagnostics. Markel took notice of Apple’s venture into the clinical trial sector through the launch of the ResearchKit platform [5], which provides clinical researchers with tools to build clinical trial apps that can be accessed by iPhone users. Markel sees this type of technology as potentially transformative, and he took note of a comment made by Alan Yeung, medical director of Stanford Cardiovascular Heath and an investigator involved with the ResearchKit cardiovascular app. 11,000 iPhone users signed up for this app within the first 24 hours of its launch, and Yueng said to Bloomberg News, “To get 10,000 people enrolled in a medical study, it would take a year and 50 medical centers around the country. That’s the power of the phone” [6]. This approach to clinical research is not without controversy, as observers are concerned iPhone-based apps can result in a biased selection of users. Others have reservations about the privacy of clinical trial data collected from these sources.

Personal health data are being collected and shared at record volumes in this era of smart phones and wearable devices. Although the openness of this data is up for debate, more intimate and personal data have caused even greater contention in recent years. Open Humans is an open online platform that asks users to share their genomes and other personal information, which can be accessed by anyone who signs into the website, and is intended to make more data available to researchers [7]. Markel sees this sort of platform as a powerful and rich source of data for computational biologists, but it’s not without controversy. Although users can share their data using an anonymous profile, the data may contain enough unique information to reveal an individual’s identity, which could have unintended consequences.

The success and wide acceptance of these open data projects will impact how the general public sees computational biology as a field, and it may take decades for the public to decide how data should be shared. Nonetheless, it’s an exciting time for computational biology according to Markel, as he sees aspects of the field coming into daily life more and has witnessed how researchers in industry labs have leveraged the power of computation.

## Ruth Nussinov

**Fig 5:**
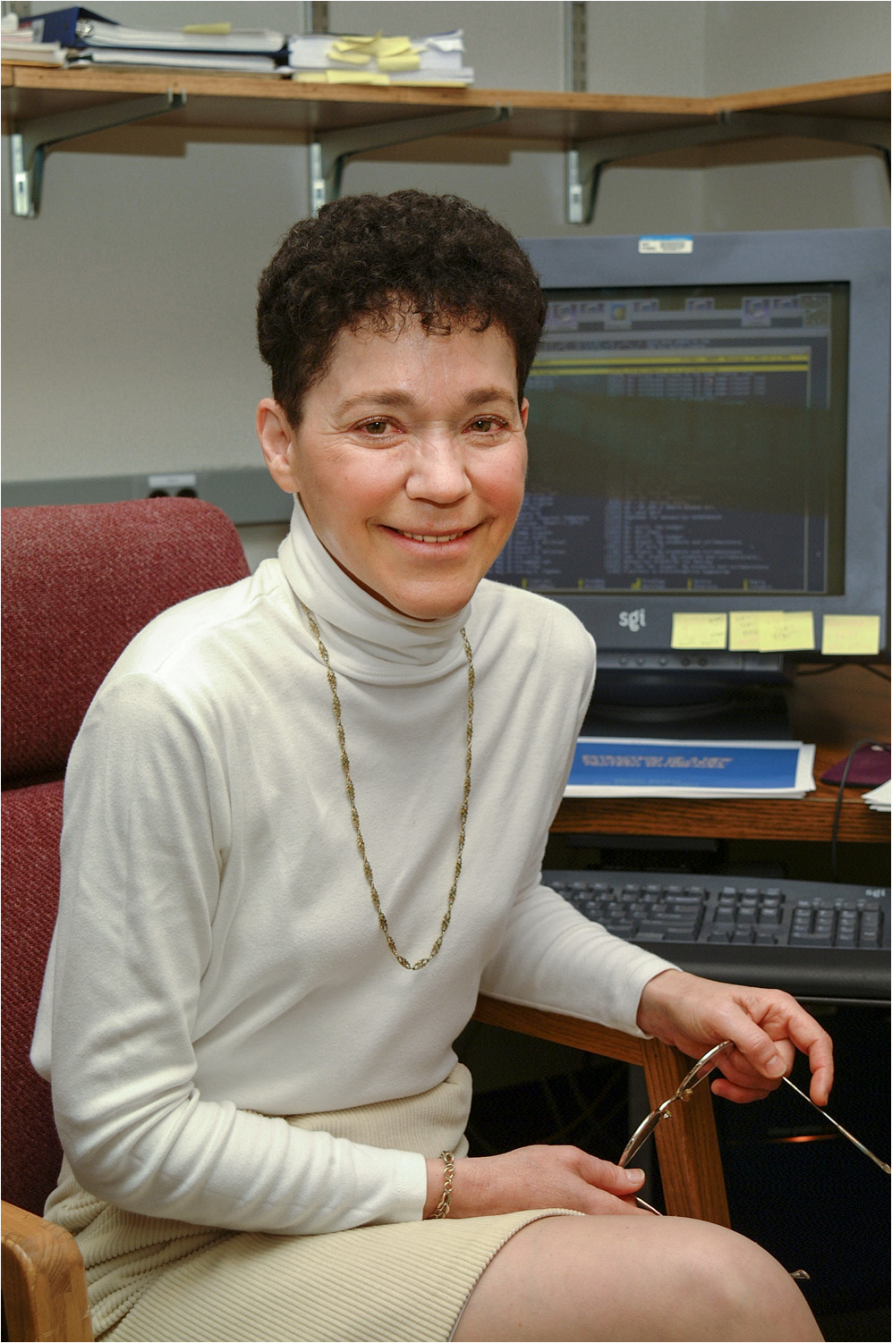
Ruth Nussinov. Senior investigator and head of computational structural biology group, Laboratory of Experimental Immunology, Cancer and Inflammation Program, NCI, NIH, and professor in the School of Medicine, Tel Aviv University.

Ruth Nussinov (Fig 5) heads the computational structural biology group in the Laboratory of Experimental Immunology at the National Cancer Institute (NCI)/NIH and is editor-in-chief of *PLOS Computational Biology*. She earned a PhD in biochemistry from Rutgers University and did her postdoctoral training at the Weizmann Institute. She has spent her career working as a computational biologist and is a pioneer of DNA sequence analysis and RNA structure prediction. Nussinov began her training at a time when the term “computational biology” was poorly understood by biologists and mathematicians and no formal training programs that combined computer science, mathematics, and biology existed. In 1985, Nussinov accepted a position as an associate professor at the Tel Aviv University Medical School, where she began an independent research program. She recalled a conversation with a dean at the school. He said, “Ruth, what are you? A mathematician?” To his chagrin and befuddlement, she replied, “No, I’m a biologist, a computational biologist.” Now computational biology is one of the hottest and fastest growing fields in biology, and training programs are in high demand.

As editor-in-chief of *PLOS Computational Biology*, Nussinov has gained a unique perspective of the field. The breadth of expertise across the journal’s Editorial Board and community of peer reviewers is vast because computational biology as a discipline is so broad; it seems to cover everything.

The vibrancy of the field is clear to Nussinov. “I think we can say it is a field that is very much alive and at the forefront of the sciences,” she said, “It reflects the fact that biology has been shifting from descriptive to a quantitative science.” Nussinov also acknowledges that computation-driven research can’t move forward without a strong relationship with experimental biology. “Computational biology is strongly tied to experiments, and experimental biology is becoming more quantitative. More and more studies provide quantitation, and this type of information is essential for making comparisons across experiments.” As a student, Nussinov recalls reading papers about transcription in which transcription levels were classified as + or ++ and were clearly subjective estimates of transcription levels. Now transcript levels are quantified with exquisite sensitivity using real-time PCR.

In spite of biology’s shift toward quantitation, Nussinov recognizes some of the field’s limitations. She has worked closely with mathematicians throughout her career, and she recalls one conversation with an algorithm development mathematician. He was trying to understand all the parameters of her experiments, and she kept saying, “It depends.” She, like many biologists, is all too familiar with the numerous variables and experimental conditions that come along with seemingly messy biology experiments, and computational biologists spend much of their time contending with this issue. New technologies, especially those based in biophysics, have contributed to improvements in the quality of data used for quantitation, but some variability will always exist.

Nussinov feels that data storage and organization are critical issues facing the future of computational biology. “Data is accumulating fast, and it is extremely diverse.” One of the challenges she sees is how does the community organize the data. She said, “The data relates to populations, disease associations, symptoms, therapeutic drugs, and more. How do you organize it and make it open and shared? By disease, by countries, more isolated or less isolated areas?” These are not easy issues to address and will only become more important as data accumulate. She also considers noise to be a major issue with this data. She said, “How do you overcome the problem of noise, an inevitable problem with vast quantities of data? How do you sift through it and see real trends? You still need cross validation.”

Nussinov believes that some of the major challenges facing computational biologists deal with developing modes of analyses that can validate or negate common beliefs or expectations, uncover unknown trends, obtain insights into fundamental processes, and exploit this information to improve predictions and design. For these, the computational biologist needs data that are openly accessible, shared software, computational power, and importantly, in-depth understanding.

In the end, Nussinov sees immeasurable value in fostering collaborations between experimental and computational biologists. “Experimentalists can’t check all possible models. Computation can provide leads, and experiments can check it. That is the ideal scenario.”

## Janet Thornton

**Fig 6:**
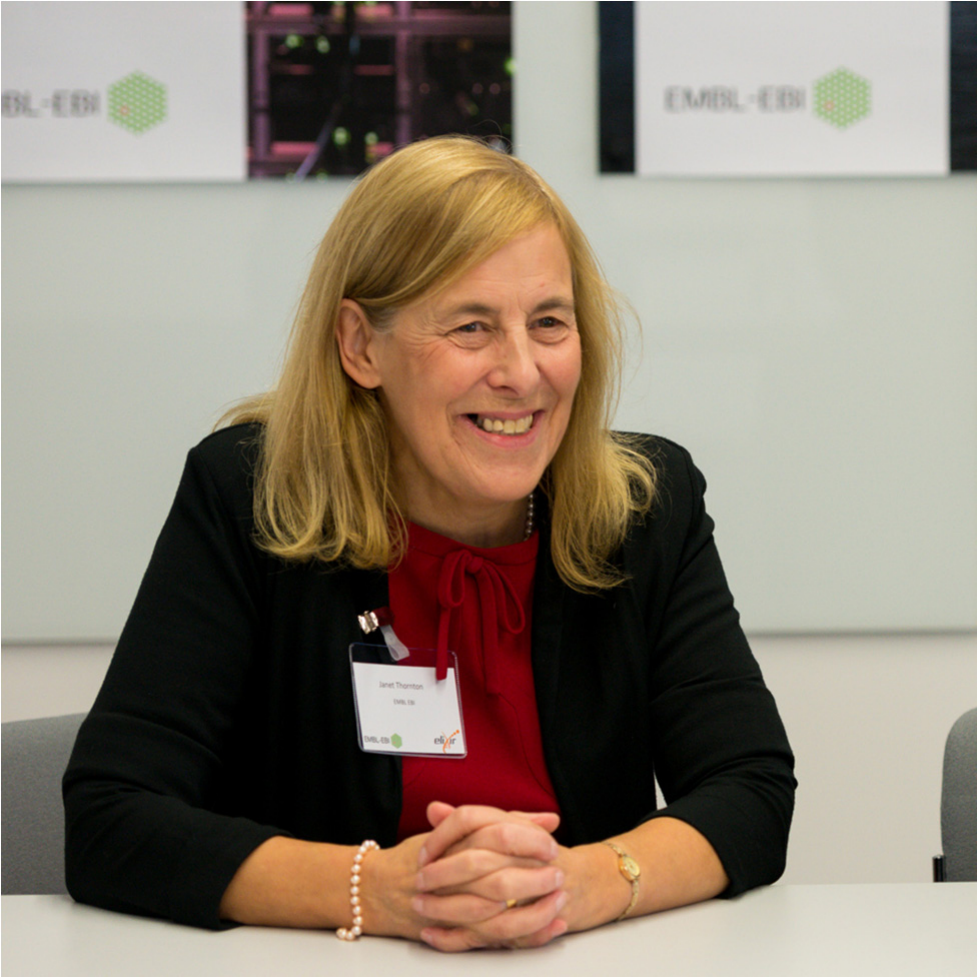
Janet Thornton. Senior scientist and outgoing director of EMBL-EBI.

Janet Thornton (Fig 6) has spent her research career studying protein structure and is considered a leading researcher in the field of structural bioinformatics. She pursued her PhD in biophysics in the 1970s, when very little information existed on protein structure and nucleotide sequences [8]. Thornton’s early research career at Oxford included using protein sequences to predict structure, and this type of research marked the earliest beginnings of bioinformatics. She became the director of the European Molecular Biology Laboratory–European Bioinformatics Institute (EMBL-EBI) in 2001, just as genomics and bioinformatics were growing rapidly, and her institution maintained valuable bioinformatics databases with data from throughout Europe. EBI also developed a thriving bioinformatics research community.

Thornton’s experiences as a structural biologist and EBI director have given her an exclusive viewpoint on the evolution of computational biology and bioinformatics from its infancy to the present day. She considers developments in five different areas to have been critical to the progress of computational biology:

1. *Development of new methods for new data-generating technologies (next-generation sequencing*, *proteomics/metabolomics*, *genome-wide association studies [GWAS]*, *and image processing)*. Without these methods, the new technologies would have been useless and interpretation of the data impossible.
2. *Development of methods in systems biology*.
3. *Ontology development and text mining*. This area is fundamental to everything in computational biology. Defining the ontologies and the science behind them will ultimately allow for the data integration and comparison needed to understand the biology of life. The opportunities presented by open literature and open data cannot be underestimated, and new methods for text mining are being developed.
4. *Algorithm development*. The effectiveness and efficiency of old algorithms (sequence alignment and protein folding) is constantly being refined, and new algorithms are being developed alongside new technologies.
5. *Technical development*. New methods for handling, validating, transferring, and storing data at all levels are under development, and cloud computing for the biological sciences is emerging.

Thornton reflected on some of the most interesting observations and results to come out of the increasingly diverse corpus of computational biology research—in particular, the use of genomics to identify how microbes evolve during an epidemic, genomic approaches to understanding human evolution, GWAS studies to discern how genetic variants impact disease, the discovery of the breadth of the microbiome and how bacterial populations interact and influence each other, the use of electronic health records to extract clinical data, and the observation that regulatory processes evolve relatively quickly in comparison to protein sequences and structures.

Thornton’s research has changed over time as bioinformatics tools and algorithms improved and protein data flooded the databases. She explained, “The evolution of protein function, especially understanding how the majority of enzyme functions have developed during their evolution from other functions, has been helped by new sequence data for the construction of better [phylogenetic] trees that reveal yet more interesting changes in function.” New algorithms developed in our group have changed the way we compare enzyme functions and have made it quantitative rather than qualitative.” Thornton is also studying how variants affect structure and function, and the 1,000 genomes data have greatly enhanced this work. She said, “The major difference in this area is new data. We can now look at germ-line changes in many individuals. Relationships to diseases are emerging, and many new paradigms will be revealed with 100,000 genomes from individuals with rare diseases.”

The convergence of computational and experimental biology is already underway, and Thornton considers that several pressing biological questions can only be addressed by combining these approaches—in particular, in areas such as building predictive models of the cell, organelles, and organs, understanding aging, designing enzymes, and improving drug design and target validation.

Thornton considers one of the biggest challenges facing computational biology, and potentially hindering these areas of research, is sharing data, especially medical data. She also believes that the computational biology community must make engagement of medical professionals and the public a top priority. She said, “This is really important. At EMBL-EBI, we are training medical professionals in bioinformatics, working on more and more public engagement, which is a huge challenge to do across Europe, especially with limited funds, and we are training scientists to do more public engagement.” It seems clear that computational biology will become a part of everyday life, especially in medicine, and these efforts are critical for gaining support from the medical community and the greater public.

## Conclusion

The thoughts shared by these accomplished computational biologists make it clear that biology is becoming a data science, and future breakthroughs will depend on strong collaborations between experimental and computational biologists. Biologists will need to adapt to the data-driven nature of the discipline, and the training of future researchers is likely to reflect these changes as well. Aspects of computational biology are integrating into all levels of medicine and health care. Medical professionals as well as the public need to be well informed and educated about these changes in order to realize the full potential of this new frontier in medicine without fear of the technological advances.
